# Preferential Re-Replication of *Drosophila* Heterochromatin in the Absence of Geminin

**DOI:** 10.1371/journal.pgen.1001112

**Published:** 2010-09-09

**Authors:** Queying Ding, David M. MacAlpine

**Affiliations:** Department of Pharmacology and Cancer Biology, Duke University Medical Center, Durham, North Carolina, United States of America; The Hospital for Sick Children and University of Toronto, Canada

## Abstract

To ensure genomic integrity, the genome must be duplicated exactly once per cell cycle. Disruption of replication licensing mechanisms may lead to re-replication and genomic instability. Cdt1, also known as Double-parked (Dup) in *Drosophila*, is a key regulator of the assembly of the pre-replicative complex (pre-RC) and its activity is strictly limited to G1 by multiple mechanisms including Cul4-Ddb1 mediated proteolysis and inhibition by geminin. We assayed the genomic consequences of disregulating the replication licensing mechanisms by RNAi depletion of geminin. We found that not all origins of replication were sensitive to geminin depletion and that heterochromatic sequences were preferentially re-replicated in the absence of licensing mechanisms. The preferential re-activation of heterochromatic origins of replication was unexpected because these are typically the last sequences to be duplicated in a normal cell cycle. We found that the re-replication of heterochromatin was regulated not at the level of pre-RC activation, but rather by the formation of the pre-RC. Unlike the global assembly of the pre-RC that occurs throughout the genome in G1, in the absence of geminin, limited pre-RC assembly was restricted to the heterochromatin by elevated cyclin A-CDK activity. These results suggest that there are chromatin and cell cycle specific controls that regulate the re-assembly of the pre-RC outside of G1.

## Author Summary

Catastrophic consequences may occur if the cell fails to either completely copy the genome or if it duplicates some regions of the genome more than once in a cell cycle. The cell must coordinate thousands of DNA replication start sites (origins) to ensure that the entire genome is copied and that no replication origin is activated more than once in a cell cycle. The cell accomplishes this coordination by confining the selection and activation of replication origins to discrete phases of the cell cycle. Start sites can only be selected or ‘licensed’ for DNA replication in G1 and similarly, they can only be activated for the initiation of DNA replication in S phase. Disruption of the mechanisms that regulate this ‘licensing’ process have been shown to result in extensive re-replication, genomic instability and tumorigenesis in a variety of eukaryotic systems. Here we use genomic approaches in *Drosophila* to identify which origins of replication are susceptible to re-initiation of DNA replication in the absence of replication licensing controls. Unexpectedly, we find that sequences in the heterochromatin, which were thought to contain only inefficient origins of replication, are preferentially re-replicated. These results provide insights into how origins of replication are selected and regulated in distinct chromatin environments to maintain genomic stability.

## Introduction

The precise duplication of the genome is a fundamental requirement to maintain genomic integrity. Eukaryotic cells employ a tightly regulated origin licensing system to ensure that each origin of DNA replication is activated once and only once during S phase [1]. Failure to properly license replication origins will result in either re-initation of DNA replication or underreplication, and may contribute to genomic instability. A critical feature of this licensing system is that the assembly of the pre-replicative complex (pre-RC) at origins of replication is separated from replication initiation by strictly limiting each process to distinct phases of the cell cycle [1], [2]. In G1, when CDK levels are low, Cdc6 and Cdt1 function to load the replicative helicase complex (MCMs) at ORC binding sites to form the pre-RC. The pre-RC is subsequently activated in S phase by cyclin and Dbf4 dependent kinase (CDK and DDK) activities which results in the loading of DNA polymerases and the initiation of bi-directional DNA replication.

Re-initiation of DNA replication within the same cell cycle is prevented by multiple redundant mechanisms that prevent the re-assembly of the pre-RC outside of G1. For example, in *S. cerevisiae* and *S. pombe*, pre-RC re-assembly is prevented by CDK dependent activities which alter the phosphorylation and localization of Cdc6 and the MCM proteins [3]–[5]. In metazoans, the primary mechanism by which re-replication is prevented is the down regulation of Cdt1 activity by either the proteosome mediated degradation of Cdt1 or by the inhibitory binding of geminin to Cdt1 [6]. Geminin is present only in higher eukaryotes and its expression is limited to the S, G2 and M phases in proliferating metazoan cells [7], [8]. Geminin binds and sequesters Cdt1 in an inactive complex that cannot recruit the MCMs thereby suppressing origin licensing [9], [10]. The degradation of geminin in late M phase releases Cdt1 from geminin and allows the reformation of the pre-RC in the subsequent G1.

Disruption of licensing mechanisms leads to re-replication in a variety of model organisms. The overexpression of Cdt1 by itself or in combination with Cdc6 results in re-replication in p53 deficient human cancer cell lines [11]. Similarly, Cdt1 overexpression or geminin depletion has also been demonstrated to result in re-replication in *C. elegans* [12] and *Drosophila* [13], [14]. In contrast, Cdt1 overexpression is insufficient to induce re-replication in yeast; instead, the simultaneous mutation of multiple pre-RC components is required to override the licensing control mechanisms [15], [16].

Regardless of the exact mechanism(s) that prevent re-replication, a common feature among eukaryotes is that not all sequences are equally susceptible to re-initiation of DNA replication. In human cancer cell lines, re-replication induced by Cdt1 overexpression or Cdt1/Cdc6 co-overexpression occurs in early replicating regions of the euchromatin [11], whereas genome-wide studies in both S. *cerevisiae* and S. *pombe* have shown that re-initiating origins of replication are distributed throughout the genome with increased re-replication at subtelomeric sequences [17]–[19]. These studies also suggest the intrinsic ability of an origin to re-initiate DNA replication is regulated both at the level of pre-RC assembly and activation [18].

The deregulation of replication licensing leads to DNA damage, checkpoint activation, aneuploidy and genomic instability [11], [20]–[22]. We sought to better understand how origins of replication are selected and activated in the absence of replication licensing controls. Specifically, we have depleted geminin from *Drosophila* Kc167 cells and assessed the consequences of re-replication using genome-wide approaches. We found that the pericentromeric heterochromatin was preferentially re-replicated in the absence of geminin. Re-replication of the heterochromatin was due to the dynamic re-assembly of a limited number of pre-RCs whose assembly was restricted to the heterochromatin by cyclin A-CDK activity. These results provide insights into how cell cycle controls and chromatin environment facilitate the selection and regulation of origins of replication.

## Results

### Geminin depletion induces DNA re-replication

The increase in DNA content observed in the absence of geminin in both *Drosophila* [13] and human cell lines [20] does not follow precise genome-unit integer steps, but rather represents a broad continuum of DNA content greater than 4N. The non-integer increase of DNA copy number observed in geminin depleted cells suggests that certain sequences may have a differential capacity to re-initiate DNA replication. We sought to characterize the susceptibility of unique sequences in the *Drosophila* genome to re-initiate DNA replication in the absence of geminin.

**Figure 1:**
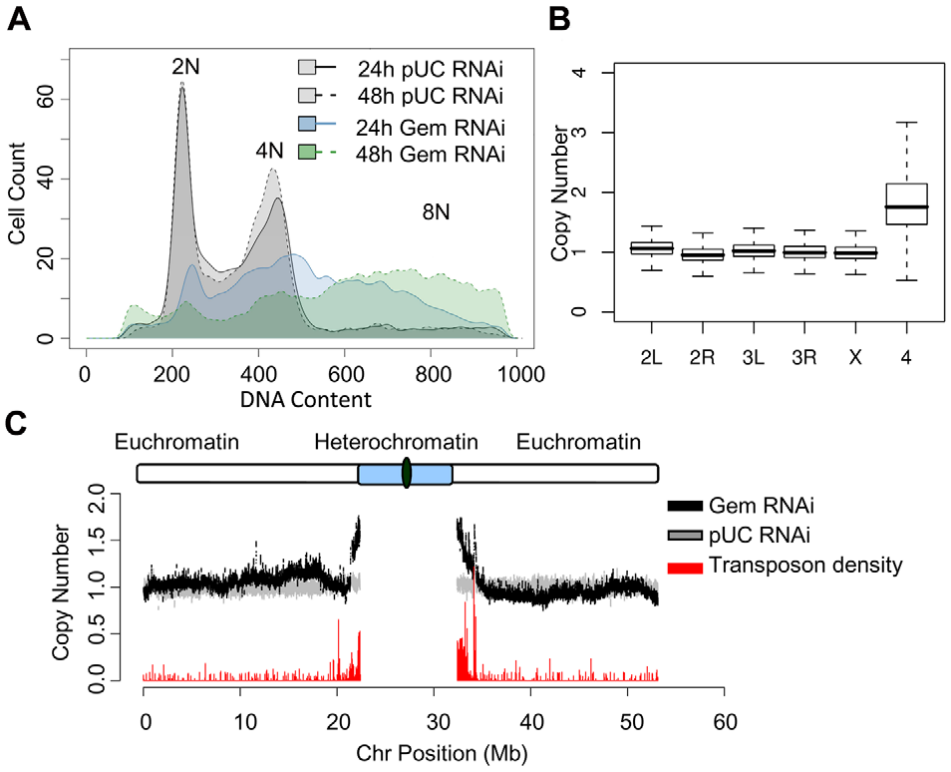
Geminin depletion results in increased ploidy. (A) Histogram of DNA content determined by flow cytometry for cells treated with nonspecific pUC control dsRNA for 24 (gray; solid outline) and 48 hours (gray; dashed outline) or geminin dsRNA for 24 (blue; solid outline) and 48 (green; dashed outline) hours. (B) Boxplot of the relative copy number of DNA from geminin depleted cells at 48 hours versus DNA from control cells for individual array probes grouped by chromosome. The heterochromatic 4th chromosome is at a significantly elevated copy number (p<1×10^−16^). (C) The relative copy number for array probes following 48 hours of geminin depletion (black) as a function of chromosomal position for the left and right arms of *Drosophila* chromosome 2. The relative copy number comparing two independent populations of control cells is shown in gray. Transposon density (fraction of sequence covered by transposable elements) is indicated by the red histogram at the bottom of the plot.

RNA interference (RNAi) was used to reduce the expression of geminin in *Drosophila* Kc167 cells. Asynchronous cells were treated with dsRNA targeting either geminin or a non-specific control sequence derived from the plasmid pUC119 (pUC). Geminin protein levels were depleted in a time dependent manner in the cells treated with geminin dsRNA (Figure S1). In contrast, geminin levels were not perturbed by treatment with pUC dsRNA. As previously reported [13], [23], we observed a decrease in the stability of Dup, the *Drosophila* Cdt1 homolog, in the absence of geminin. Consistent with published data [13], flow cytometry of DNA content revealed that geminin deficiency resulted in the inappropriate re-replication of the genome. After only 24 hours of treatment with geminin dsRNA, a large population of cells (>50%) contained more than 4N DNA content and by 48 hours of treatment, many of the cells had a DNA content greater than 8N (Figure 1A).

### Pericentromeric heterochromatin is preferentially re-replicated

To identify sequences prone to re-replication in geminin depleted Kc167 cells, we utilized comparative genomic hybridization (CGH) to directly measure the relative change in DNA copy number generated as a result of re-replication. In this assay, DNA from independent biological replicates was harvested from either geminin or pUC dsRNA treated cells, differentially labeled with Cy5 and Cy3 conjugated dUTP, and hybridized to custom genomic tiling microarrays containing unique probes spanning the sequenced *Drosophila* euchromatin. The log ratio of Cy5 to Cy3 signal served as a proxy for the relative copy number difference between geminin depleted and control cells.

Geminin depletion resulted in strikingly elevated levels of the fourth chromosome (Figure 1B). The *Drosophila* fourth chromosome is unique because of its small size, high transposon density and heterochromatic nature [24]. Given the distinct chromatin environment of the fourth chromosome, we sought to more closely examine copy number throughout the remaining *Drosophila* chromosomes. We analyzed the relative copy number of DNA from geminin depleted cells versus control DNA across each of the chromosomes by plotting the moving average of DNA copy number as a function of chromosome position. The relative copy number for the large majority of euchromatic sequences along the chromosome arms of geminin depleted cells was constant and equal to one. However, as the sequences approach the pericentromeric heterochromatin on chromosome 2, 3 and X, the copy number increased sharply, indicating that DNA sequences in these regions have re-replicated (Figure 1C and Figure S2, black).

In *Drosophila*, repressive chromatin is marked by the trimethylation of histone H3 on lysine residue 9 (H3K9me3) in the constitutive pericentromeric heterochromatin and by trimethylation of histone H3 on lysine 27 (H3K27me3) at polycomb repressed sequences [25], [26]. The elevated copy number we observe for chromosome 4 and the sequences adjacent to the heterochromatin for each of the remaining chromosomes suggests that the constitutive heterochromatin is being preferentially re-replicated in the absence of geminin. These sequences also exhibit an increased density of transposable elements (Figure 1C and Figure S2, red). We examined the extent of re-replication for sequences marked by either H3K9me3 or H3K27me3 using data generated by the modENCODE consortium [27]. These genomic datasets describe the genome-wide location of lysine trimethylation on histone H3 using nearly identical growth conditions (including serum) as our own experiments. We found that re-replication in the absence of geminin was specific for the constitutive heterochromatin marked by H3K9me3 (Figure S3).

Because the heterochromatic sequences and those near heterochromatin have a reduced sequence complexity, we wanted to ensure that the increase in DNA copy number was not an artifact of the array hybridization. As a control, we hybridized independent samples of control DNA versus itself. The relative copy number of the control DNA was constant across each of the chromosome arms (Figure 1C and Figure S2, gray). To account for any potential variation in the copy number estimates due to the asynchronous cell cycle distribution of the control cells, we also compared by CGH analysis DNA from geminin depleted cells to cells arrested in G2/M by colcemid treatment. This analysis revealed a copy number increase for heterochromatic sequences that was indistinguishable from our prior results (Figure S4). Finally, to further validate our copy number estimation from the genomic microarrays, we tested seven different loci throughout the euchromatin and heterochromatin of chromosome 3R by quantitative PCR (qPCR). The qPCR results confirmed the heterochromatin specific 2–2.5 fold increase in DNA content number we observed by the genomic tiling arrays (Figure S5).

**Figure 2:**
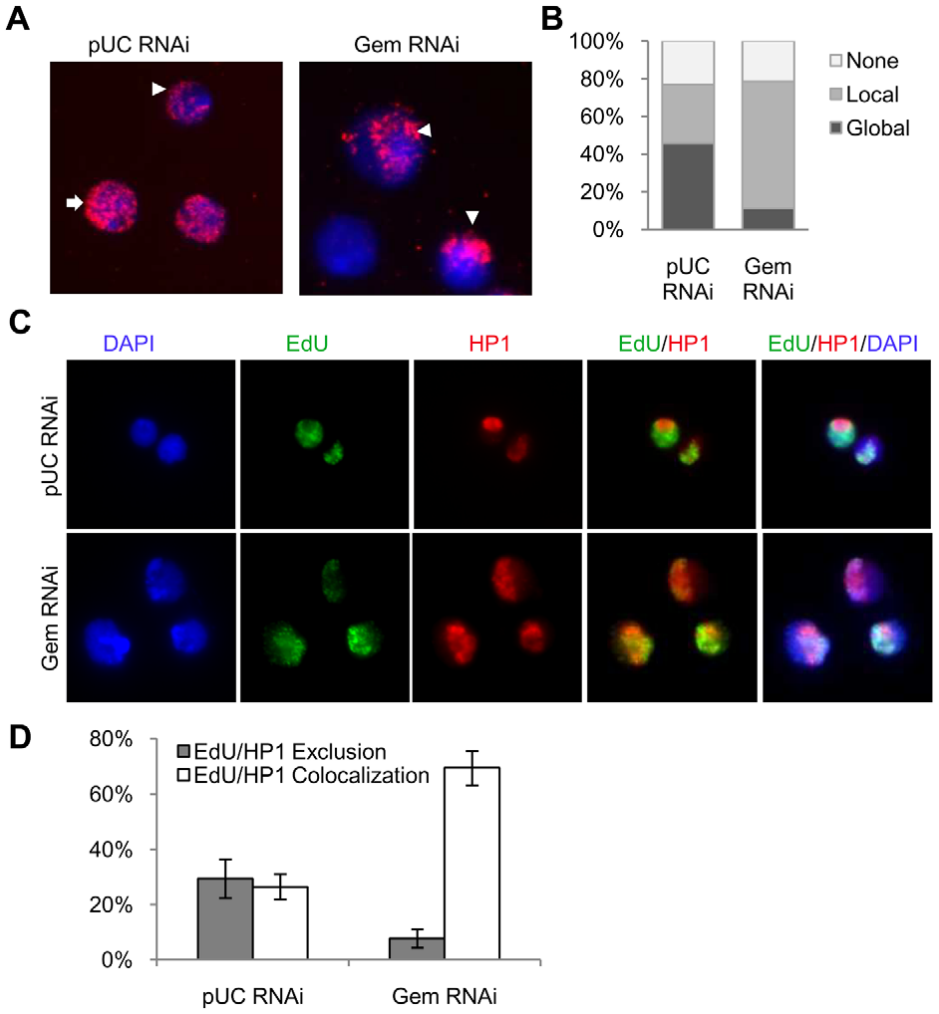
Re-replication is specific to pericentromeric heterochromatin. (A) Fluorescence microscopy of cells after a 4 hour BrdU pulse at 18 hours post RNAi treatment with control (pUC) or geminin dsRNA. BrdU labeled sequences were detected with a rat anti-BrdU antibody (red) and DNA was counterstained with DAPI (blue). BrdU staining patterns were classified as ‘global’ (arrow), ‘local’ (arrowhead) or ‘none’. (B) Distribution of the different BrdU incorporation patterns. At least 200 cells were counted from three independent experiments. The distribution of BrdU incorporation patterns was significantly different between control cells and geminin depleted cells (p<1×10^−16^). (C) Immunofluorescence of control and geminin depleted cells following a 30 min pulse of EdU at 24 hours post RNAi. EdU and HP1 were detected with Alexa Fluor 488-azide (green) and rabbit anti-HP1 antibody (red), respectively, and DNA was counterstained with DAPI (blue). (D) Quantification of EdU and HP1 localization patterns in control and geminin depleted cells with error bars indicating standard error. At least 200 cells were counted in three independent experiments. The distribution of EdU/HP1 localization patterns was significantly different between control cells and geminin depleted cells (p<1×10^−16^).

Our genomic analysis indicated that there was increased copy number of sequences adjacent to the pericentromeric heterochromatin; however, our initial genomic tiling arrays lacked representative probes from this region of the genome. To further identify which regions of the genome were being re-replicated in the absence of geminin, we assessed the cytological location of active replication by immunofluorescence. Actively replicating sequences were labeled by the incorporation of 5-bromo-2-deoxyuridine (BrdU). BrdU was added to the medium for a 4 hour window at 18 hours post RNAi treatment, at which time re-replication is clearly detectable by FACS in geminin depleted cells (data not shown). The cells treated with pUC dsRNA exhibited multiple distinct patterns of BrdU incorporation, including: no BrdU incorporation, BrdU incorporation all over the nucleus, and BrdU incorporation localized to a small region at the nuclear periphery (Figure 2A). These distinct patterns of BrdU incorporation were classified as either ‘none’, ‘global’, or ‘local’, respectively (Figure 2B). These patterns likely represent cells in different stages of the cell cycle. For example, cells with no BrdU staining (none) were likely in G2-M-G1 of the cell cycle, cells with limited BrdU in a confined region at the nuclear periphery (local) were in late S phase, and finally those cells with BrdU incorporation throughout the nucleus (global) represent cells in early to mid S phase [28]. In contrast, almost 90% of BrdU staining geminin depleted cells exhibited the ‘local’ BrdU incorporation pattern in a small region of the nucleus (Figure 2A and 2B). These results were consistent with re-replication being confined to a specific region of the genome.

The ‘local’ pattern of BrdU incorporation in the nucleus, the detection of increased copy number near the pericentromeric heterochromatin and the increased copy number for sequences marked by H3K9me3 in geminin depleted cells suggested that the pericentromeric heterochromatin was being preferentially re-replicated in the absence of geminin. In *Drosophila*, the pericentromeric heterochromatin is enriched for HP1, a heterochromatic protein that interacts with H3K9me3 [26]. To confirm that re-replication was localized to the heterochromatin, we used immunofluorescence to simultaneously detect newly synthesized DNA and HP1 (Figure 2C). Control and geminin depleted cells were pulse labeled with 5-ethynyl-2-deoxyuridine (EdU), for 30 minutes at either 24 or 48 hours post RNAi. EdU was used for these experiments because unlike BrdU, the denaturation of DNA was not required for detection. In control cells we observe that EdU is either specifically co-localized with HP1 (actively replicating heterochromatin) or that it is excluded from the HP1 staining regions of the genome (actively replicating euchromatin) (Figure 2C and 2D). In contrast, geminin depleted cells undergoing re-replication almost exclusively exhibit co-localization of EdU and HP1, consistent with the preferential re-replication of the heterochromatin. Together with the array data, these results show that the majority of DNA synthesis occurring in geminin depleted cells is restricted to heterochromatic regions of the *Drosophila* genome.

### Re-replication is not coupled with late replication

Origins of replication are activated in S phase with a characteristic timing and efficiency. Chromatin structure is thought to play a role in establishing the temporal order of origin activation [29]. For example, active transcription and histone acetylation have been positively correlated with early replication in a variety of model organisms [30]–[34]. Similarly, poorly transcribed regions of the genome, often marked by repressive chromatin modifications, are typically the last sequences to be duplicated in S phase [31], [35]. Our genomic and cytological results are paradoxical because they suggest that in the absence of geminin, the initiation of DNA replication is most efficient in the pericentromeric heterochromatin marked by H3K9 methylation and HP1. These findings imply that either late replicating sequences are more prone to re-replication or that the observed re-replication is a function of chromatin environment.

**Figure 3:**
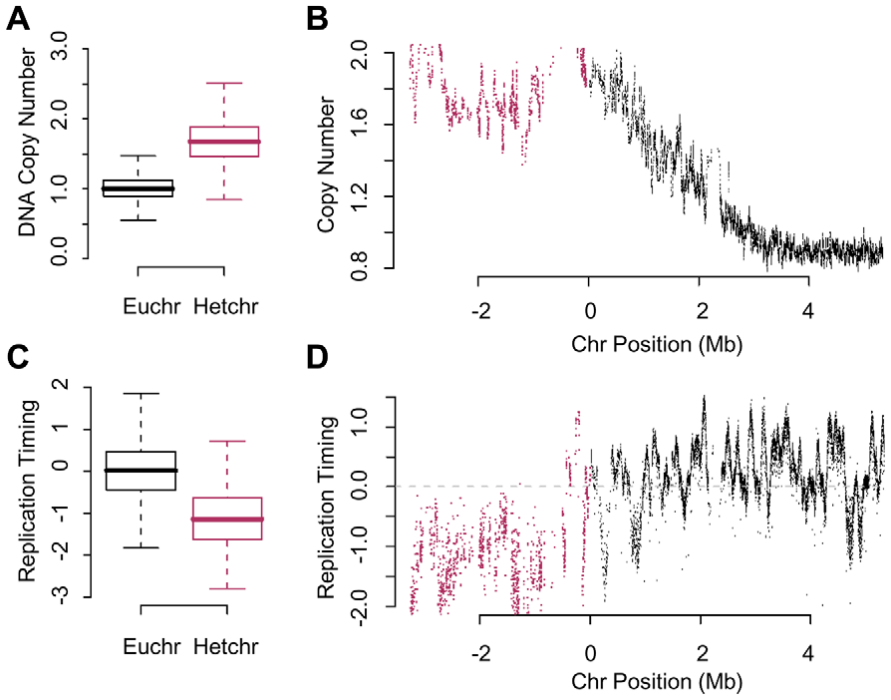
Re-replication is dependent on chromatin environment not time of replication. (A) Boxplot of the relative copy number between geminin depleted and control cells for array probes in the euchromatin or heterochromatin. The heterochromatin is at a signficantly higher copy number (p<1×10^−16^). (B) The relative copy number for array probes in the vicinity of pericentromeric heterochromatin on the right arm of chromosome 2 following geminin RNAi treatment for 48 hours (euchromatin, black; heterochromatin, red). (C) Boxplot of the relative time of replication for sequences in the euchromatin or heterochromatin from normal mitotic cells. The heterochromatin is signficantly later replicating than the euchromatin (p<1×10^−16^). (D) Replication timing values as a function of chromosomal position for unique sequence probes in the vicinity of the pericentric heterochromatin on the right arm of chromosome 2 (euchromatin, black; heterochromatin, red).

To determine if susceptibility to re-replication was a function of late replicating sequences or a heterochromatic chromatin enviroment, we examined the extent of re-replication and the relative time of replication evident in heterochromatic sequences using a custom array design [36] that, in addition to the euchromatic sequences, contained all of the unique *Drosophila* heterochromatic sequences [37]. DNA was isolated from control and geminin depleted cells and hybridized to the new genomic arrays. Statistical analysis of the relative probe levels between control and geminin depleted cells revealed a clear increase in copy number for those probes located in the annotated heterochromatin (Figure 3A; p<1×10^−16^). Analysis of the data relative to chromosome position revelead a constant one and a half to two-fold increase in ploidy in the heterochromatin (red) relative to the euchromatin (black), with a shallow transition from heterochromatin to euchromatin, as shown for a representative region of the right arm of chromosome 2 (Figure 3B). Importantly, as our previous array data indicated, re-replication and the increase in DNA copy number was limited to the heterochromatin and adjacent sequences.

The normal mitotic replication timing of the heterochromatin was examined by differentially labeling early and late replicating sequences from synchronized cells [35], [36]. Analysis of global replication timing values from either euchromatic or heterochromatic sequences revealed that the heterochromatin replicated significantly later than the euchromatin (Figure 3C; p<1×10^−16^). We did not detect the presence of efficient clusters of mitotic replication origins in the heterochromatin which would be evident as a continuum of enriched probes culminating in a clear and defined peak, as shown for the right arm of chromosome 2 (Figure 3D). Importantly, late replication does not appear to be a determinant of re-replication as there are many late replicating regions in the euchromatin that do not appear to re-replicate as shown for a representative region of the genome (Figure 3B and 3D). These data argue that susceptibility to re-initiation of DNA replication is distinct from the mechanisms that regulate replication timing.

### Limited pre-RC assembly in the absence of geminin

Prior to initiation of DNA replication, the pre-RC must be assembled at origins of replication. Typically, low CDK levels and the absence of geminin allow for the global assembly of pre-RCs in G1 [2]. We hypothesized that re-replication in the absence of geminin may be limited to the heterochromatin by restricting either pre-RC assembly or activation to these sequences. For example, in the absence of geminin the re-assembly of the pre-RC may occur almost exclusively in the heterochromatin. Alternatively, there may be a global re-assembly of the pre-RC throughout the *Drosophila* genome, but initiation of DNA replication is strictly limited to the heterochromatin. To differentiate between these two models, we assessed the re-loading of MCMs onto the DNA by chromatin fractionation and immunofluorescence.

**Figure 4:**
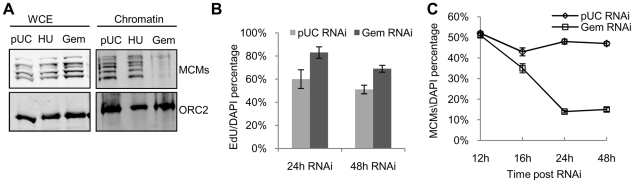
Limited pre-RC re-assembly occurs in the absence of geminin. (A) Chromatin from control RNAi (pUC), G1/S arrested (HU), and geminin depleted re-replicating cells (Gem) was fractionated biochemically and immunoblotted for MCMs and ORC2. Levels of ORC and MCMs are also shown for whole cell extracts (WCE). (B) Fraction of control or geminin depleted cells exhibiting active DNA synthesis (EdU incorporation), with error bars indicating standard error. DNA synthesis was determined by pulsing the cells for 30 minutes with EdU at the given time points. At least 200 cells were counted in three independent experiments. (C) Time course of the fraction of control (open diamond) or geminin depleted (open square) cells exhibiting nuclear MCM localization following RNAi treatment for the indicated duration, with error bars indicating standard error. At least 200 cells were counted in three independent experiments.

We first examined the global levels of chromatin-associated MCMs by chromatin fractionation. Chromatin was prepared from pUC dsRNA treated cells (pUC), cells arrested with hydroxyurea (HU), and cells treated with geminin dsRNA (Gem) for 24 hours. ORC remained associated with the chromatin fraction in all samples. In contrast, MCM association with chromatin was only observed in pUC RNAi cells and cells arrested at the G1/S border by treatment with HU. A marked reduction in MCM levels was observed on the chromatin prepared from geminin depleted cells while total MCM levels were not affected by geminin depletion (Figure 4A). Despite the reduction in chromatin-associated MCM levels in the geminin depleted cells, a significant number of cells (60%–85%) exhibited active DNA synthesis as measured by a 30 minute EdU pulse (Figure 4B). Thus, cells undergoing re-replication are able to synthesize DNA with a reduced complement of MCM proteins.

To gain further insights into the mechanism of MCM reloading, we examined MCM loading and DNA synthesis at the single cell level. The association of MCMs with chromatin was assessed by immunofluorescence of cells treated with a mild detergent and salt prior to fixation. The mild salt extraction ensures that only the loaded and active MCMs remain bound to the chromatin [38], [39]. We found that the fraction of MCM positive staining cells gradually decreased during geminin RNAi treatment. At 24 hours following geminin depletion, less than 20% of the cells had detectable MCM staining (Figure 4C); however, more than 80% of the cells exhibited active DNA synthesis. Even after 48 hours of geminin depletion, nearly 70% of the cells exhibited DNA synthesis following a 30 minute EdU pulse (Figure 4B). We consistently observed that the number of geminin depleted cells that were actively synthesizing DNA was much higher than the number of cells exhibiting detectable staining for chromatin-associated MCMs. Together, these data suggest that in the absence of geminin, pre-RC assembly is inefficient and that minimal levels of MCM re-assembly are sufficient for un-regulated DNA replication.

### pre-RC re-assembly at heterochromatin

**Figure 5:**
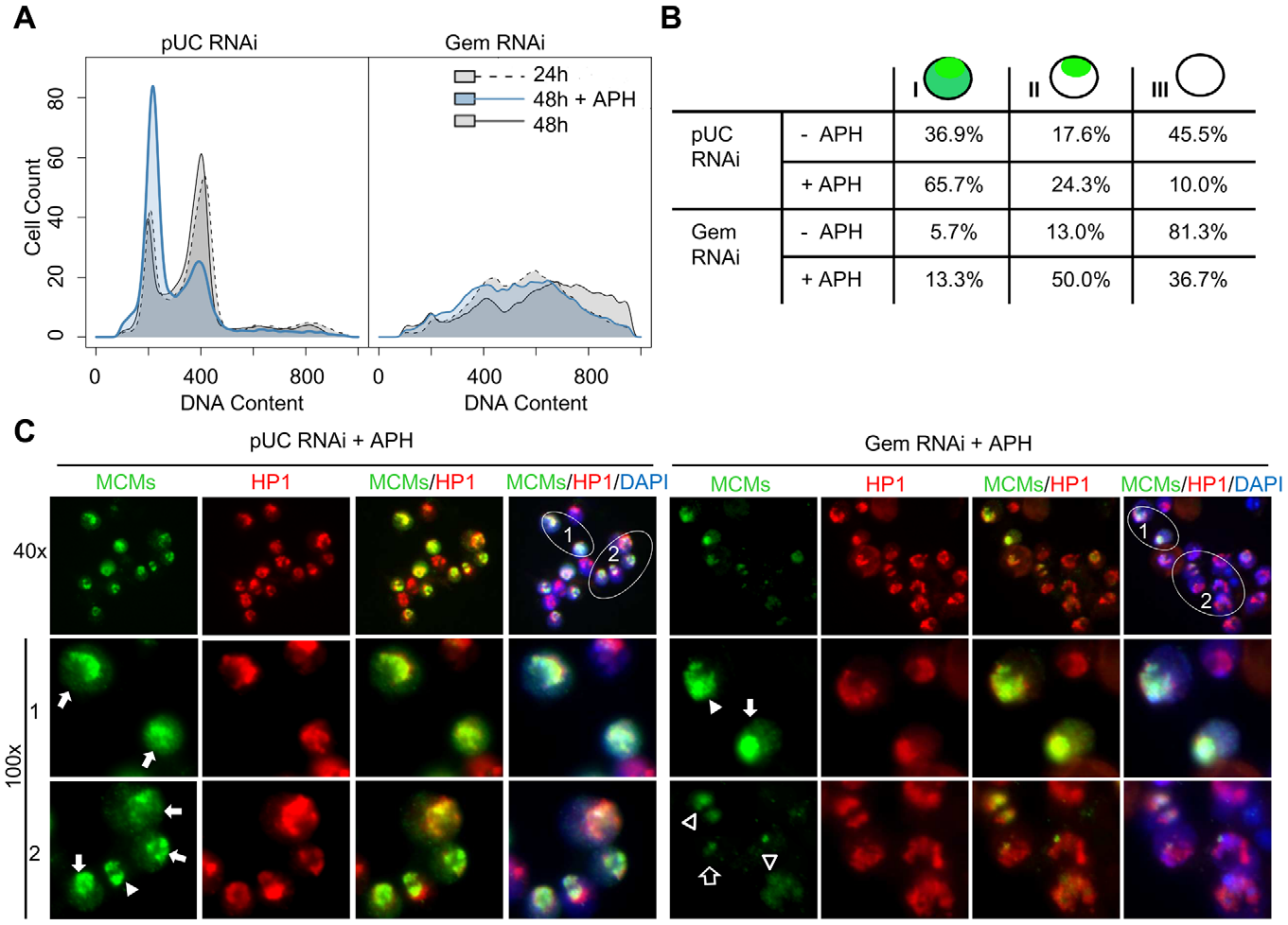
MCMs are preferentially loaded onto heterochromatin in the absence of geminin. (A) Flow cytometry analysis of DNA content for cells treated with nonspecific pUC or geminin RNAi for 24 hours (dotted outline; gray), 48 hours (solid outline; gray) or 48 hours with aphidicolin (APH) added at 24 hours (solid outline; blue). (B) Quantification of MCM localization patterns. MCM staining was classified into 3 types: type I - MCM localization throughout the nucleus, type II - MCM localization to a small portion of the nucleus (heterochromatin), and type III - no significant MCM localization observed. The numbers under each staining pattern represent the percentage of nuclei with that particular pattern. At least 200 cells were counted in three independent experiments. The distribution of MCM localization patterns in geminin depleted cells treated with aphidicolin was signficantly different from control cells treated with aphidcolin (p<1×10^−16^) and similarily, geminin depleted cells were significantly different from geminin depleted cells treated with aphidicolin (p<1×10^−16^). (C) Low and high magnification examples of type I (arrow) and II (arrow head) MCM staining patterns in control and geminin depleted cells treated with aphidicolin. The MCMs, HP1 and DAPI are shown in green, red and blue, respectively. The type II pattern of MCM localization in geminin depleted cells often exhibited heterogeneous staining with faint signal over the entire HP1 region (open arrowhead) or partial overlap with HP1 (open arrow).

The low levels of MCMs which are sufficient for un-licensed re-initiation of DNA replication in the absence of geminin made it difficult to assess whether the re-assembly of the pre-RC was limited to the heterochromatin or occurred throughout the *Drosophila* genome. We hypothesized that during re-replication the re-assembly of the pre-RC might be labile with the MCMs exhibiting a short half life on the DNA. In order to extend the half-life of the MCMs on chromatin, we used aphidicolin to inhibit DNA polymerase and arrest any active replication forks. DNA synthesis was inhibited in both control and geminin depleted cells at 24 hours post RNAi treatment by the addition of aphidicolin (Figure 5A). The majority of control cells arrest with an early S-phase FACS profile upon exposure to aphidicolin. Similarily, aphidicolin inhibited additional re-replication in the absence of geminin and the cells arrest with a DNA content profile indistinguishable from that of cells depleted of geminin for 24 hours.

In normally dividing cells, the chromatin association of MCM proteins changes thoughout the cell cycle [40]–[43]. During G1, the MCMs are loaded onto the chromatin to form the pre-RC and to license replication origins for entry into S phase. As DNA replication progresses through S phase, the MCMs are displaced from the DNA by the passage of the replication fork [40], [41]. Thus, during G1 and early to mid S phase the MCMs are localized throughout the entire nucleus, and by late S phase they only remain associated with late replicating sequences. We classified the untreated control cells into three categories based on their MCM localization patterns (Figure 5B). In 37% of the control cells, the MCMs localized throughout the nucleus with increased staining at the heterochromatic region marked by HP1(type I). We interpret these cells to be in G1 and early to mid S phase. In contrast, 18% of cells appeared to be in late S phase with MCM staining limited to heterochromatin (type II) while the remaining 46% of cells were devoid of nuclear MCM staining (type III) and likely in G2 or M phase. The distributions of cells in each phase of the cell cycle were consistent with the FACS profiles (Figure 5A). Treatment with aphidicolin altered the cell cycle distribution of control cells with the majority of cells accumulating at the G1/S transition and exhibiting the type I staining pattern (Figure 5B).

Treatment of geminin depleted cells with aphidicolin inhibited further re-replication and resulted in a clear increase in chromatin-associated MCM levels as evidenced by the increase in the number of cells with positive MCM staining (type I and II, from 19% to 63%). Unlike control cells where the majority of MCMs were localized throughout the nucleus upon exposure to aphidicolin (Figure 5C arrow), the predominant localization pattern in the geminin depleted cells was at the heterochromatin, co-incident with HP1 staining (Figure 5B and 5C). In contrast to the strong uniform heterochromatic staining we observe in control cells and a few untreated geminin depleted cells, aphidicolin treatment of geminin depleted cells often resulted in a weak and heterogeneous staining of the MCMs throughout the heterochromatin. For example, there was light staining localized with a subfraction of HP1 (Figure 5C, open arrow) as well as faint and diffuse staining which completely colocalized with HP1 (Figure 5C, open arrowhead). This weak and relatively heterogenous staining was only detected in geminin depleted cells treated with aphidicolin and may represent a random selection of heterochromatic origins of replication on a cell by cell basis.

### Cyclin A-CDK activity restricts pre-RC formation to the heterochromatin

**Figure 6:**
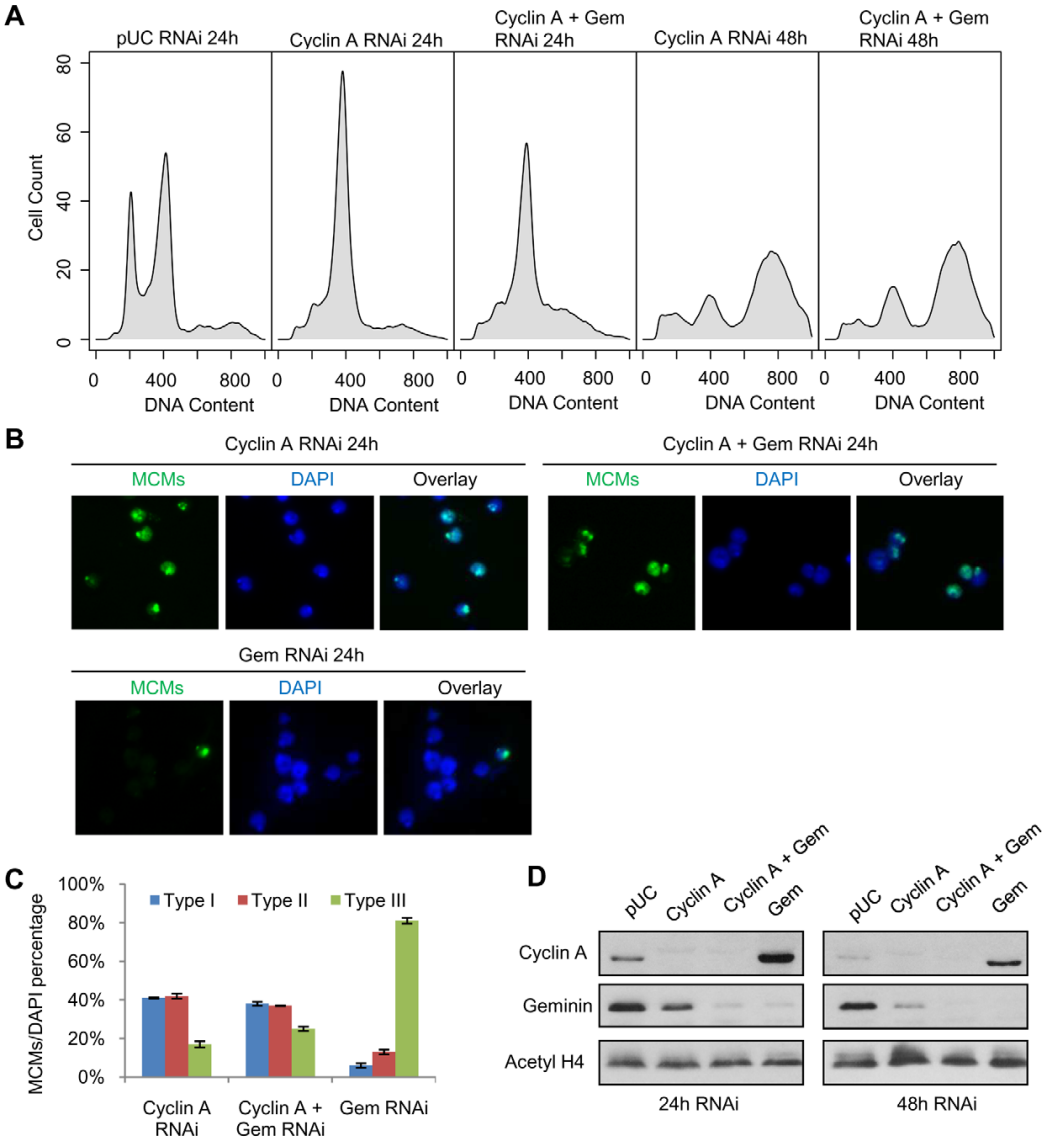
Cyclin A - CDK activity restricts pre-RC formation to the heterochromatin. (A) Histogram of DNA content for cells treated with pUC dsRNA, cyclin A dsRNA or cyclin A and geminin dsRNAs for 24 hours and 48 hours. (B) Immunostaining of chromatin-associated MCMs at 24 hours post cyclin A, cyclin A and geminin, or geminin RNAi treatment. (C) Quantification of the different MCM localization patterns as described in Figure 5, with error bars indicating standard error. At least 200 cells were counted from three independent experiments. The distribution of MCM localization patterns in geminin depleted cells was significantly different from cyclin A depleted cells (p<1×10^−16^) and cells simultaneously depleted of geminin and cyclin A (p<1×10^−16^). (D) Immunoblot analysis of cyclin A and geminin levels following the individual and co-RNAi depletion experiments.

Prior studies in human cells indicated that cyclin A-CDK activity stimulates re-replication in the presence of excess Cdt1 or Cdc6 [11]. Knockdown experiments in *Drosophila* cells showed cyclin A silencing suppressed the partial re-replication induced by geminin depletion [13]. We sought to investigate whether cyclin A-CDK activity plays a role in restricting pre-RC assembly to the *Drosophila* heterochromatin. As reported previously [13], we also observed that depletion of cyclin A by RNAi arrests cells at G2/M, and after a delay of approximately 24 hours, the cells initiate a complete endoreduplication of their genome (Figure 6A, compare panel 2 and 4). Importantly, in the absence of both geminin and cyclin A, there is only limited re-replication at 24 hours (panel 3) and, similiar to the cyclin A depleted cells, we observe a complete reduplication of the genome by 48 hours (panel 5). The suppression of partial re-replication by cyclin A depletion is not due to inefficient depletion of geminin in the cyclin A co-knockdown experiment (Figure 6D). Presumably, the decreased CDK activity associated with the cyclin A depletion allows for the genome-wide re-assembly of the pre-RC which would facilitate the complete reduplication of the genome. We examined the distribution of chromatin-associated MCMs in cells depleted for cyclin A, geminin, or cyclin A and geminin. In the absence of cyclin A, the majority of cells (∼80%) exhibited MCM loading on the chromatin 24 hours prior to the endoreduplication (Figure 6B and 6C). An equal number of cells with type I (throughout the nucleus) and type II (restricted to the heterochromatin) MCM localization patterns were observed. Similar results were obtained for cells depleted of both geminin and cyclin A (Figure 6B and 6C). This is in sharp contrast to the almost complete absence of chromatin associated MCMs observed in cells depleted only for geminin (Figure 5B and Figure 6C). Together, these results suggest that pre-RC re-assembly may not be specifically targeted to the heterochromatin, but rather the re-assembly of the pre-RC in euchromatin is inhibited by persistent cyclin A-CDK activity.

## Discussion

Maintenance of constant genome ploidy is critical for eukaryotic organisms. If unchecked, disruption of the mechanisms that tightly couple DNA replication with the cell cycle may result in re-replication, aneuploidy and genomic instability. We have found that perturbation of Dup activity by geminin depletion results in the preferential re-replication of heterochromatic sequences in the *Drosophila* genome. Re-replication was limited to pericentromeric heterochromatic sequences which are marked by HP1 and H3K9 methylation. Euchromatin, including gene-poor late replicating sequences and polycomb repressed sequences, was resistant to re-replication. In the absence of geminin, a minimal complement of MCMs assembled on the chromatin was sufficent for re-replication. These findings suggest that the 2–3 fold increase in ploidy we observed was regulated by the specific re-assembly and activation of the heterochromatic pre-RCs.

The preferential re-replication of heterochromatic sequences in the absence of licensing controls was particularly striking given the established view that repressive chromatin environments are inhibitory to efficient origin activation [44]. Classic experiments have clearly demonstrated that the heterochromatin in *Drosophila* and other ogranisms is the last region of the genome to be duplicated during S phase [45]. The complex nature of the heterochromatic sequences has hampered detailed analysis of the replication program in this part of the genome and it remains possible that the heterochromatin may be populated by a very limited number of ultra-efficient origins of replication. These may be required to ensure that the heterochromatin is duplicated in a timely manner at the end of S phase and that in the absence of licensing controls these origins are preferentially activated. Our genomic data did not identify any robust origins of replication in the heterochromatin that were consistently used across the cell population. Similarly, we found that the bulk of heterochromatin was re-replicated to similar ploidy levels suggesting that origin re-activation in the absence of geminin is a stochastic process.

Analysis of total DNA ploidy by FACS revealed that the majority of cells exhibited a DNA content greater than 8N following geminin depletion, suggesting geminin depleted cells had increased their total DNA content by at least 2-fold over that of G2 cells. The heterochromatin constitutes a minimum of 30% of the *Drosophila* genome [46]. Assuming that re-replication is specific to the heterochromatin, a 4.5 fold increase in heterochromatic DNA content would be sufficient to account for the increase in DNA ploidy we observe. However, we consistently observed, by multiple methods, only a 2–2.5 fold increase in heterochromatic DNA content (Figure 1, Figure S2 and S4). We speculated that the highly repetitive non-unique heterochromatic sequences might be preferentially re-replicated to higher ploidy levels. We tested this hypothesis by examining the genomic abundance of the 1.688 satellite DNA which accounts for 4% of the *Drosophila* genome [47]. Again, we only observed a 2–2.5 fold increase in the bulk levels of the 1.688 satellite DNA (Figure S6). It is clear that the heterochromatin is preferentially re-replicated in the absence of geminin; however, we are unable to rule out the possibility that a limited amount of stochastic re-replication is also occurring in the euchromatin.

In higher eukaryotes, there are many more MCM complexes loaded onto the chromatin in G1 than are required to complete an unperturbed S phase [48]–[51]. Although these excess MCMs are not required for completion of a normal S phase, they are critical for protecting the cell from genomic instability during replication stress [52]. However, when we deplete geminin, we only observe a minimal complement of MCMs being re-loaded onto heterochromatic DNA. These results suggest that there is not a global re-assembly of the pre-RC throughout the genome as occurs in G1 and that limiting amounts of MCMs are sufficient for the greater than two-fold increase in ploidy we observe. The MCMs appear to be transiently associated with the heterochromatin as inhibition of DNA re-replication with aphidicolin results in an increase in detectable MCMs. We propose that in the absence of geminin, the MCMs are loaded onto heterochromatic sequences and that these pre-RCs are immediately activated for initiation of DNA replication.

In *Drosophila*, Dup activity is downregulated after origin firing through multiple mechanisms including Cul4-Ddb1 mediated proteolysis in S phase and inhibition by geminin during S, G2 and mitosis. Furthermore, we (Figure S1) and others [13], [23] have reported that Dup/Cdt1 is degraded in the absence of geminin. Thus, geminin is only one factor that negatively regulates Dup/Cdt1 and its depletion may be insufficient to induce genome-wide re-replication. Therefore, the deregulation of geminin and Dup/Cdt1 may have distinct effects on replication control. This may, in part, explain the differences in sequences and chromatin environment which are preferentially targeted for re-replication in human and *Drosophila* cell lines. In human cell lines, Cdt1 overexpression led to the preferential re-replication of early replicating sequences [11], while in *Drosophila* cell lines, geminin depletion leads to the preferential activation of heterochromatic origins of replication. Future experiments will test whether Dup levels are critical for maintaining ploidy and selecting which origins are activated.

The observation that the re-replication at pericentromeric heterochromatin was not coupled with late replication timing suggests that origin selection during re-replication and the temporal control of DNA replication in S phase are regulated by distinct mechanisms. These data suggest that a key determinant of which sequences will re-initiate DNA replication is the local chromatin environment. *Drosophila* pericentromeric heterochromatin is marked by H3K9 methylation which is maintained by *Su(var)*3-9 and HP1[53], [54]. ORC has been shown to interact with HP1 and localizes to heterochromatin by immunofluorescence in both interphase and mitotic nuclei [55], [56]. It is therefore possible that the increased density of ORC in the heterochromatin may stimulate the preferential re-assembly of the pre-RC at those sequences. However, recent studies using GFP tagged ORC and live imaging did not observe an increased density of ORC at the heterochromatic regions of the genome [57].

We found that cyclin A-CDK activity regulates the re-activitation of replication origins at two levels. First, cyclin A-CDK activity is required for the large increase in ploidy observed, consistent with the known role of CDK activity in activating the pre-RC for initiation [1]. Second, cyclin A-CDK activity appears to differentially inhibit pre-RC re-assembly in the euchromatin and heterochromatin. The simultaneous depletion of both cyclin A and geminin results in the global re-assembly of the pre-RC in both euchromatin and heterochromatin (Figure 6). In contrast, depletion of only geminin results in pre-RC re-assembly specific to the heterochromatin, suggesting that cyclin A-CDK activity specifically inhibits pre-RC assembly in the euchromatin. In humans, the N-terminal domain of ORC1 contains consensus CDK phosphorylation sites which can be phosphorylated *in vitro* by cyclin A-CDK activity and may regulate the SCF/Skp2 mediated turnover of ORC1 during S phase [58]. In *Drosophila*, the ORC1 N-terminus also contains potential CDK phosphorylation sites, an additional O-box for APC mediated destruction [59], and is essential for the binding of ORC1 with HP1 [56]. We propose that the interaction between ORC1 and HP1 may protect ORC1 from inhibitory cyclin A-CDK signals or destruction by the APC, thereby differentially sensitizing heterochromatic and euchromatic origins of replication to un-licensed pre-RC assembly.

## Materials and Methods

### Cell growth and drug treatment

*Drosophila* Kc167 cells were cultured at 25°C in Schneider's Insect Cell Medium (Invitrogen) supplemented with 10% fetal bovine serum and 1% penicillin/streptomycin/glutamine (Invitrogen). DNA synthesis was inhibited by treatment with 1 mM hydroxyurea (Sigma) or 7 µM aphidicolin (Sigma).

### RNA interference

All double-stranded RNAs were generated using gene-specific PCR products (∼700bp) flanked by T7 polymerase-binding sites as templates for *in vitro* transcription reactions with the T7 RiboMax express large-scale RNA production system (Promega). Primers are available upon request. For each RNAi experiment, cells were washed with and diluted in serum-free medium to a final concentration of 2×10^6^ cells/ml. 15µg dsRNA was added per 1×10^6^cells, gently mixed, and incubated for one hour. After the incubation, 2× medium was added resulting in a final concentration of 1×10^6^ cells/ml. The cells were incubated for 1–2 days before harvesting.

### Array hybridization and data analysis

DNA was isolated as described [60]. Isolated DNA was sheared and labeled with either fluorescent Cy5- or Cy3-conjugated dUTP (GE Healthcare Bio-Sci Corp.) using Sequenase (US Biochemicals) and a random nonamer oligo (IDT). The labeled DNA was purified using Microcon YM-30 filters (Millipore) and hybridized to custom Agilent tiling microarrays overnight at 65°C. The slides were then washed and scanned as per Agilent recommendations. The Agilent generated tif files from each genomic microarray were processed and analyzed in R [61], a free software environment for statistical computing and graphics. The LIMMA package [62] from the BioConductor project [63] was used to normalize the ratio of the Cy5 and Cy3 channels across each genomic tiling array by loess normalization. Quantile normalization was used to normalize between replicate slides. The relative copy number between control and geminin depleted cells was determined by the log ratio of Cy5 and Cy3. To assess the significance of the copy number enrichment for different genome features (chromosome, heterochromatin, euchromatin, etc) a Student's t-test was utilized and p-values are indicated in the figure legends. All genomic coordinates are based on the 5.0 assembly of the *Drosophila* genome. The analysis of the replication timing data is described in MacAlpine et al., 2010.

### Data submission and genomic data

All genomic data with accompanying metadata (protocols and analysis parameters) are publicly available at the NCBI GEO data repository. The accession numbers are: GSE17279 Kc167 replication timing, GSE20781 S2 H3K27me3, GSE20794 S2 H3K9me3, and GSE20932 Kc167 re-replication CGH data.

### Western blotting and chromatin fractionation

Antibodies used in western blotting include: rat anti-geminin antibody (a gift from Helena Richardson), anti-Dup guinea pig antibody (a gift from Terry L. Orr-Weaver), anti-acetylated H4 antibody (Upstate), and mouse anti-cyclin A antibody (Developmental Studies Hybridoma Bank). All antibodies were used at a 1∶3000 dilution.

Chromatin fractionation was carried out as described [64]. Samples were analyzed by PAGE and western blotting. Primary antibodies used include anti-Orc2 at 1∶3000 and anti-MCMs (AS1.1) at 1∶100. Secondary antibodies used include Alexa Fluor 680 goat anti-rabbit IgG (Invitrogen), IRDye 800 conjugated anti-mouse IgG (Rockland Immunochemicals), both at a 1∶10000 dilution. Immunoblots were scanned using a LICOR imaging system.

### Immunofluorescence

For the BrdU incorporation experiments, cells were fixed with methanol: acetic acid 3∶1(v/v) after a 4-hour incubation with 5µg/ml BrdU (Roche). A rat anti-BrdU antibody (Abcam Inc.) was used at 1∶200 and Alexa Fluor 594 goat anti-rat secondary antibody (Invitrogen) was used at 1∶500. For the double labeling of EdU and HP1, cells were incubated in medium with 10 µM EdU for 30 minutes, treated with 0.5% Triton X-100 in PBS for one minute, fixed with 4% paraformaldehyde in PBS, then run through the EdU Click-iT reaction cocktail (Invitrogen). Cells were then stained with rabbit anti-HP1 antibody (#191, 1∶1000, a gift from Sarah Elgin) and Alexa Fluor 568 goat anti-rabbit secondary antibody (1∶500, Invitrogen). For double labeling of MCMs and HP1, cells were treated with Triton/PBS and fixed as for EdU and HP1 staining, stained with monoclonal MCM antibody (AS1.1, 1∶100) and anti-HP1 antibody (#191, 1∶1000), followed by secondary detection with Alexa Fluor 488 goat anti-mouse and Alexa Fluor 568 goat anti-rabbit antibodies (1∶500). Chi-square tests were performed for quantifications of immunofluorescence localization patterns and the p-values are shown in the corresponding figure legends.

### Quantitative PCR

The relative copy number for seven loci spanning both euchromatin and heterochromatin on chromosome 3R was examined. Specifically, three loci were located in the pericentric heterochromatin, two in the euchromatin adjacent to pericentric heterochromatin, and the remaining two in the euchromatin distant from pericentric heterochromatin. DNA isolated from control pUC dsRNA treated cells was used to generate the qPCR standard curve. Primers are available upon request. Quantitative PCR was performed using iQ SYBR Green Supermix (Bio-Rad) on Bio-Rad iQ5 Real-Time PCR Detection System.

## Supporting Information

Figure S1 Geminin depletion by RNAi results in loss of both geminin and Dup protein levels. Cells were treated with dsRNA targeting a non-specific control (pUC) or geminin for 24 or 48 hours. The abundance of geminin and Dup was assessed by immunoblotting. Acetylated H4 was used as a loading control.
(0.80 MB TIF)

Figure S2 Heterochromatin is preferentially re-replicated during geminin depletion. The copy number of all unique sequences in the *Drosophila* genome was determined by comparative genomic hybridization (CGH) using genomic tiling microarrays. Relative DNA copy number following 48 hours of treatment with geminin (black) or non-specific control dsRNA (gray) for each of the *Drosophila* chromosomes. Transposon density (fraction of sequence covered by transposable elements) is shown in red.
(0.76 MB TIF)

Figure S3 Sequences marked by H3K9me3 but not H3K27me3 are re-replicated. Boxplots of the relative copy number for sequences marked by H3K9me3 or H3K27me3 following geminin depletion by RNAi. The genome-wide mapping of H3K9me3 and H3K27me3 was performed in *Drosophila* S2 cells by the modENCODE consortium (see reference 27). Broad peaks of enrichment for H3K9me3 and H3K27me3 were defined (see GEO submission GSE20781 and GSE20794 for details) and the relative copy number for each mark in control and geminin depleted cells was analyzed by boxplots. The increase in copy number for sequences marked by H3K9me3 in geminin depleted cells was significant (p<2×10^−16^). Although there are a few specific differences in chromatin marks between the different *Drosophila* cell lines, the bulk genome-wide distributions are very similar across *Drosophila* cell lines, thus justifying the comparison of re-replication in Kc167 cells to chromatin marks in S2 cells. We also observe similar heterochromatin specific re-replication patterns in *Drosophila* S2 cells depleted for geminin (data not shown).
(2.21 MB TIF)

Figure S4 Heterochromatin is preferentially re-replicated during geminin depletion compared to cells arrested with G2 DNA content. (A) FACs analysis of cells treated with colcemid (0.5µg/ml) in the presence (middle) or absence (left) of geminin dsRNA for 48 hours. The overlay is depicted in the right panel. (B) The relative copy number for array probes following 48 hours of geminin depletion (black) as a function of chromosomal position for the left arm of *Drosophila* chromosome 2. The relative copy number comparing two independent populations of colcemid treated control cells is shown in gray.
(3.68 MB TIF)

Figure S5 Validation of copy number at seven loci following a 48h RNAi depletion of geminin. Seven loci were tested by qPCR using primer pairs designed to unique sequences in the heterochromatin and euchromatin of the right arm of chromosome 3. The copy number at each site was normalized to the copy number of site 7 which is a unique euchromatic sequence that is distant from heterochromatin. There is a 1.5–2.5 fold increase of DNA content for loci both in and proximal to the pericentric heterochromatin.
(0.06 MB TIF)

Figure S6 Copy number analysis of the 1.688 satellite DNA during geminin depletion induced re-replication. Genomic DNA was isolated from cells treated with non-specific (pUC) or geminin dsRNA for 48 hours. The bulk level of the 359bp 1.688 satellite DNA repeat, which comprises 4% of the Drosophila genome, was analyzed by digestion with the restriction enzyme SacI which linearizes the 359bp repeat unit. The first four lanes are DNA digested with the SacI enzyme from independent control and geminin RNAi experiments. The lower band is the linearized 359bp repeat of the 1.688 satellite DNA. The right four lanes are genomic DNA without digestion and serve as a loading control. Quantification of the intensity of the 359bp repeat band was performed with Image J. The first four lanes were normalized to the 359bp band of control cells treated with non-specific pUC dsRNA (pUC1), and the last four lanes were normalized to the genomic DNA of cells treated with pUC dsRNA (pUC1). The copy number of the 1.688 satellite DNA repeat was calculated as the result of the 1.688 band intensity relative to the band intensity of corresponding genomic DNA.
(2.61 MB TIF)
