# Supplementary figures and images for: Preferential Re-Replication of Drosophila Heterochromatin in the Absence of Geminin
Source: PLoS Genet. 2010 Sep 9;6(9):e1001112. doi: 10.1371/journal.pgen.1001112 (PMC2936543; doi:10.1371/journal.pgen.1001112)

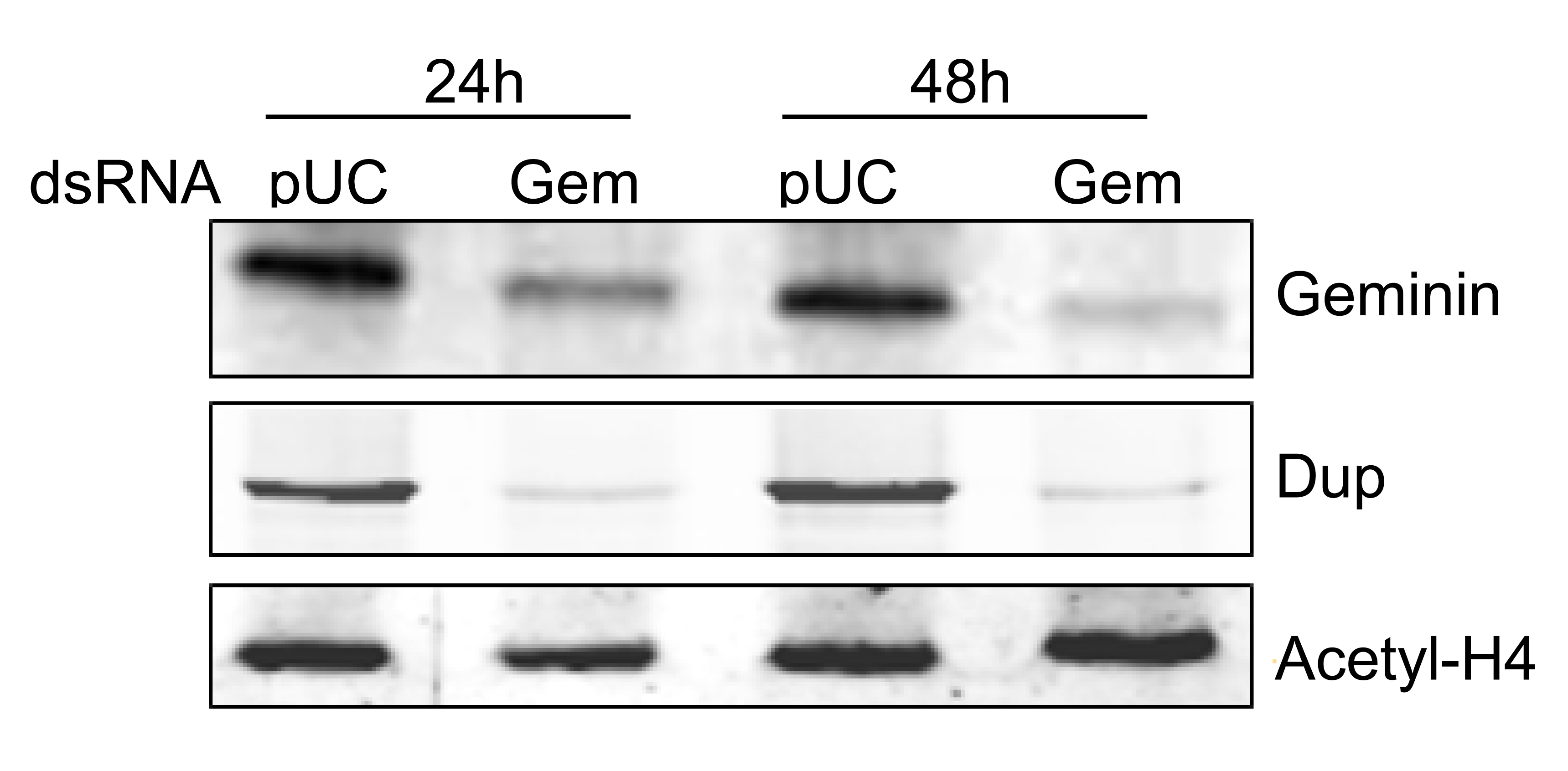

Supplement: Figure S1 — Geminin depletion by RNAi results in loss of both geminin and Dup protein levels. Cells were treated with dsRNA targeting a non-specific control (pUC) or geminin for 24 or 48 hours. The abundance of geminin and Dup was assessed by immunoblotting. Acetylated H4 was used as a loading control. (0.80 MB TIF) [file pgen.1001112.s001.tif]

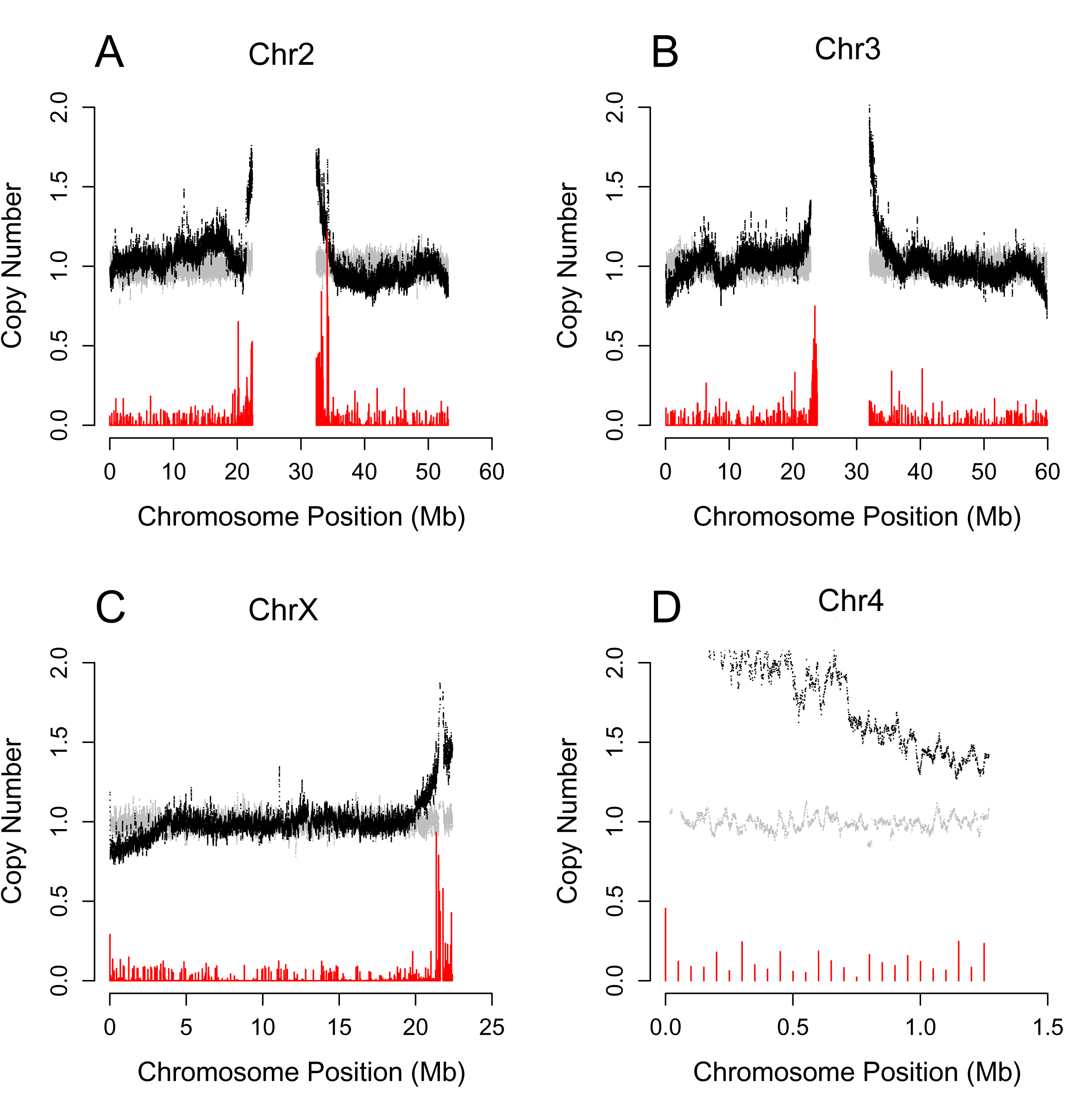

Supplement: Figure S2 — Heterochromatin is preferentially re-replicated during geminin depletion. The copy number of all unique sequences in the Drosophila genome was determined by comparative genomic hybridization (CGH) using genomic tiling microarrays. Relative DNA copy number following 48 hours of treatment with geminin (black) or non-specific control dsRNA (gray) for each of the Drosophila chromosomes. Transposon density (fraction of sequence covered by transposable elements) is shown in red. (0.76 MB TIF) [file pgen.1001112.s002.tif]

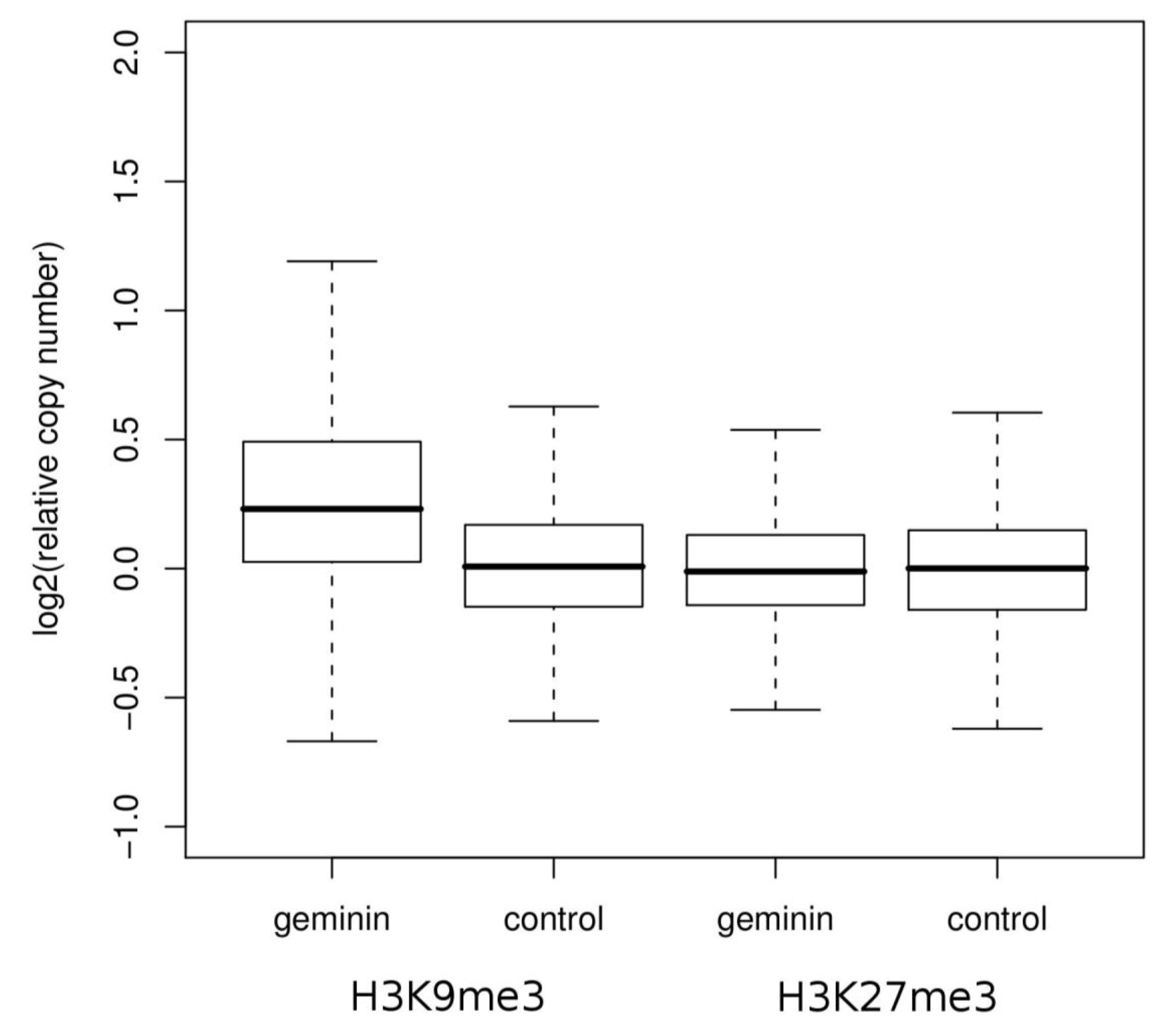

Supplement: Figure S3 — Sequences marked by H3K9me3 but not H3K27me3 are re-replicated. Boxplots of the relative copy number for sequences marked by H3K9me3 or H3K27me3 following geminin depletion by RNAi. The genome-wide mapping of H3K9me3 and H3K27me3 was performed in Drosophila S2 cells by the modENCODE consortium (see reference 27). Broad peaks of enrichment for H3K9me3 and H3K27me3 were defined (see GEO submission GSE20781 and GSE20794 for details) and the relative copy number for each mark in control and geminin depleted cells was analyzed by boxplots. The increase in copy number for sequences marked by H3K9me3 in geminin depleted cells was significant (p<2×10^−16^). Although there are a few specific differences in chromatin marks between the different Drosophila cell lines, the bulk genome-wide distributions are very similar across Drosophila cell lines, thus justifying the comparison of re-replication in Kc167 cells to chromatin marks in S2 cells. We also observe similar heterochromatin specific re-replication patterns in Drosophila S2 cells depleted for geminin (data not shown). (2.21 MB TIF) [file pgen.1001112.s003.tif]

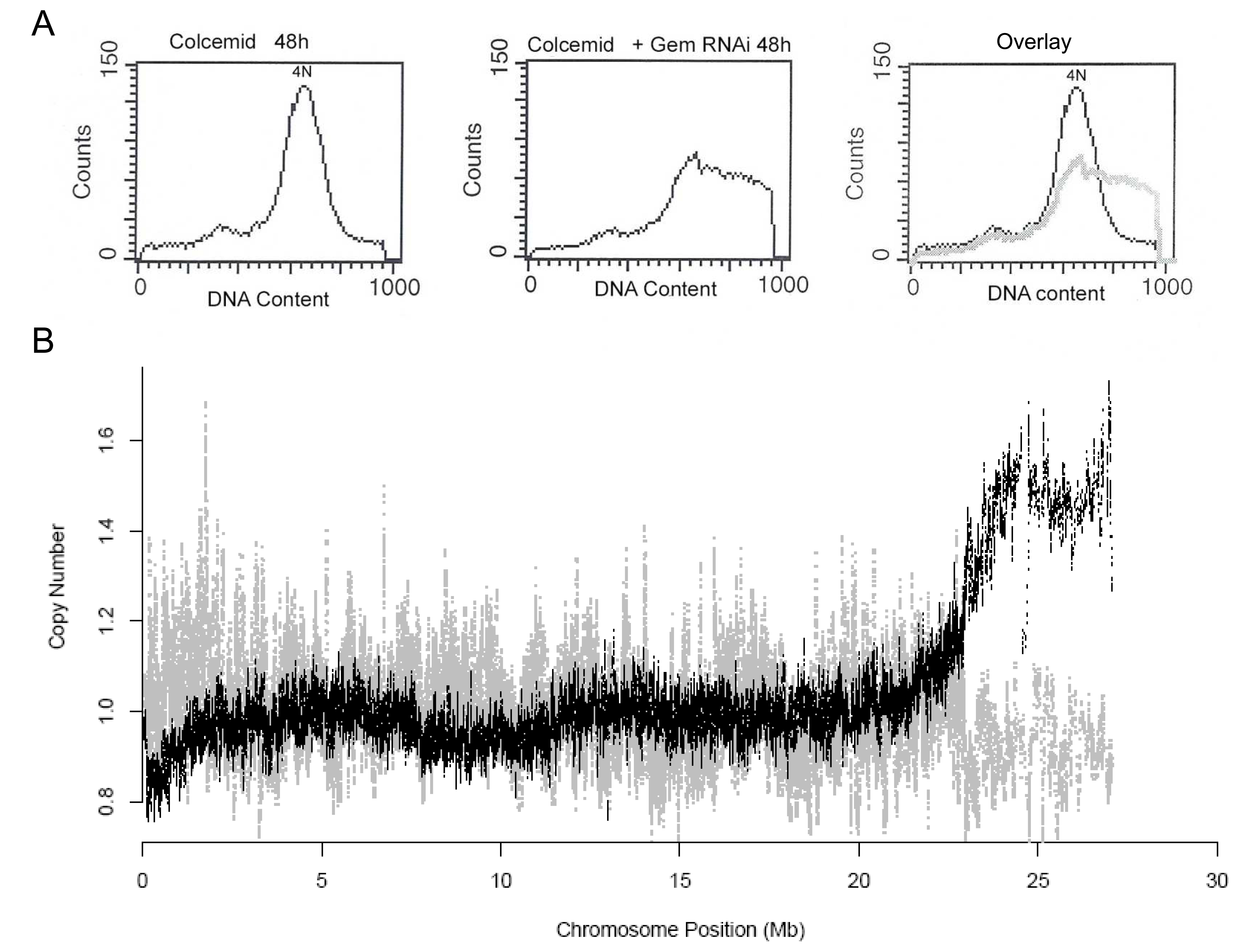

Supplement: Figure S4 — Heterochromatin is preferentially re-replicated during geminin depletion compared to cells arrested with G2 DNA content. (A) FACs analysis of cells treated with colcemid (0.5µg/ml) in the presence (middle) or absence (left) of geminin dsRNA for 48 hours. The overlay is depicted in the right panel. (B) The relative copy number for array probes following 48 hours of geminin depletion (black) as a function of chromosomal position for the left arm of Drosophila chromosome 2. The relative copy number comparing two independent populations of colcemid treated control cells is shown in gray. (3.68 MB TIF) [file pgen.1001112.s004.tif]

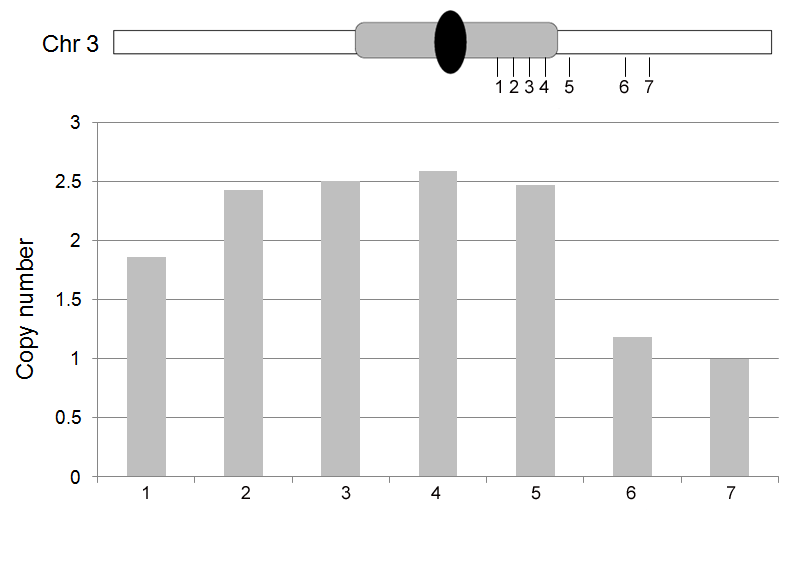

Supplement: Figure S5 — Validation of copy number at seven loci following a 48h RNAi depletion of geminin. Seven loci were tested by qPCR using primer pairs designed to unique sequences in the heterochromatin and euchromatin of the right arm of chromosome 3. The copy number at each site was normalized to the copy number of site 7 which is a unique euchromatic sequence that is distant from heterochromatin. There is a 1.5–2.5 fold increase of DNA content for loci both in and proximal to the pericentric heterochromatin. (0.06 MB TIF) [file pgen.1001112.s005.tif]

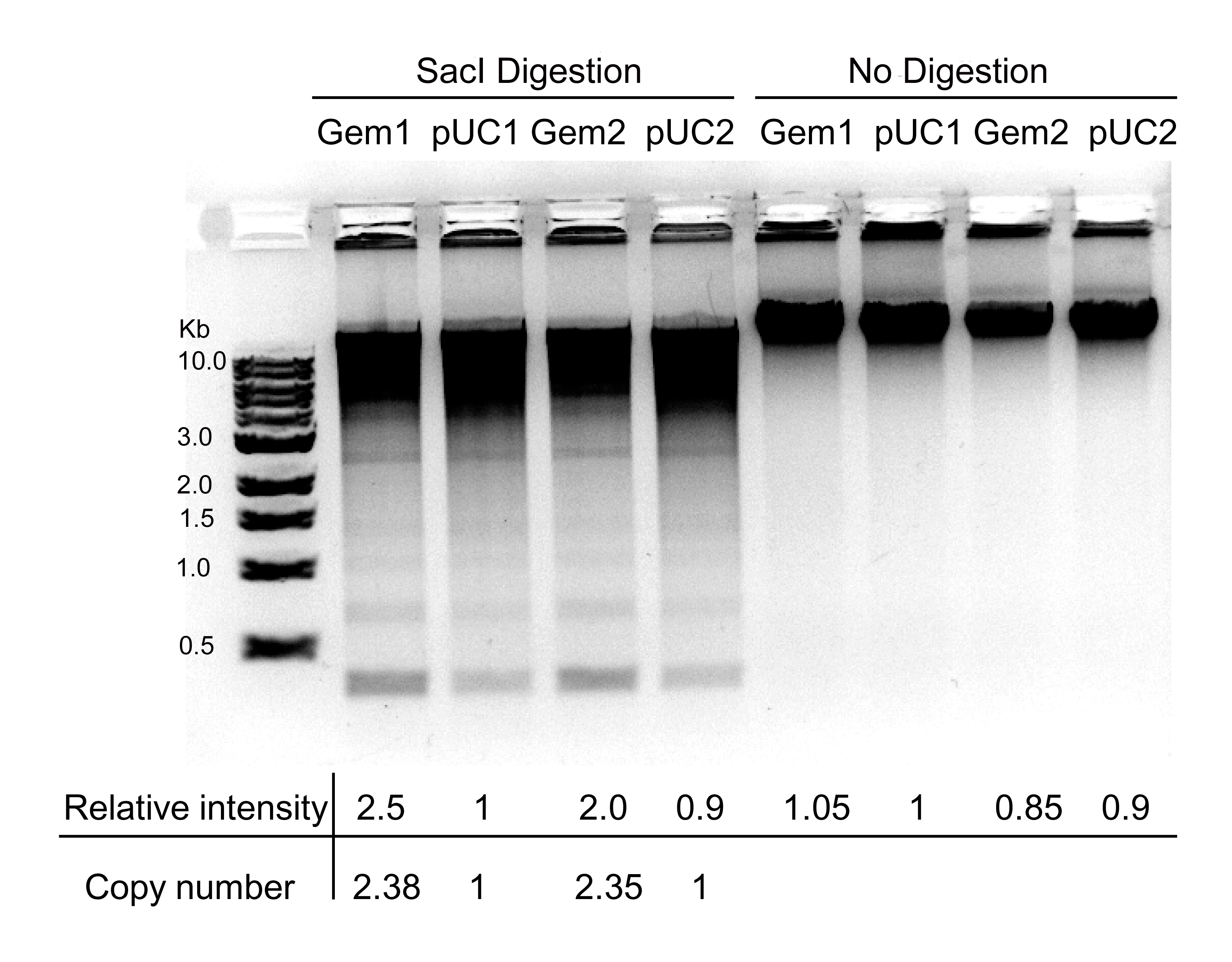

Supplement: Figure S6 — Copy number analysis of the 1.688 satellite DNA during geminin depletion induced re-replication. Genomic DNA was isolated from cells treated with non-specific (pUC) or geminin dsRNA for 48 hours. The bulk level of the 359bp 1.688 satellite DNA repeat, which comprises 4% of the Drosophila genome, was analyzed by digestion with the restriction enzyme SacI which linearizes the 359bp repeat unit. The first four lanes are DNA digested with the SacI enzyme from independent control and geminin RNAi experiments. The lower band is the linearized 359bp repeat of the 1.688 satellite DNA. The right four lanes are genomic DNA without digestion and serve as a loading control. Quantification of the intensity of the 359bp repeat band was performed with Image J. The first four lanes were normalized to the 359bp band of control cells treated with non-specific pUC dsRNA (pUC1), and the last four lanes were normalized to the genomic DNA of cells treated with pUC dsRNA (pUC1). The copy number of the 1.688 satellite DNA repeat was calculated as the result of the 1.688 band intensity relative to the band intensity of corresponding genomic DNA. (2.61 MB TIF) [file pgen.1001112.s006.tif]
